# Hydrazinocurcumin Encapsuled Nanoparticles “Re-Educate” Tumor-Associated Macrophages and Exhibit Anti-Tumor Effects on Breast Cancer Following STAT3 Suppression

**DOI:** 10.1371/journal.pone.0065896

**Published:** 2013-06-25

**Authors:** Xiwen Zhang, Wenxia Tian, Xiaozhong Cai, Xiaofei Wang, Weiqi Dang, Hao Tang, Hong Cao, Lin Wang, Tingmei Chen

**Affiliations:** Key Laboratory of Diagnostic Medicine Designated by the Ministry of Education, Chongqing Medical University, Chongqing, China; University College London, United Kingdom

## Abstract

Tumor-associated macrophages (TAMs) are essential cellular components within tumor microenvironment (TME). TAMs are educated by TME to transform to M2 polarized population, showing a M2-like phenotype, IL-10^high^, IL-12^low^, TGF-β^high^. STAT3 signaling triggers crosstalk between tumor cells and TAMs, and is crucial for the regulation of malignant progression. In our study, legumain-targeting liposomal nanoparticles (NPs) encapsulating HC were employed to suppress STAT3 activity and “re-educate” TAMs, and to investigate the effects of suppression of tumor progression *in vivo*. The results showed that TAMs treated by HC encapsuled NPs could switch to M1-like phenotype, IL-10^low^, IL-12^high^, TGF-β^low^, and the “re-educated” macrophages (M1-like macrophages) considerably demonstrated opposite effect of M2-like macrophages, especially the induction of 4T1 cells migration and invasion *in vitro*, and suppression of tumor growth, angiogenesis and metastasis *in vivo*. These data indicated that inhibition of STAT3 activity of TAMs by HC-NPs was able to reverse their phenotype and could regulate their crosstalk between tumor cells and TAMs in order to suppress tumor progression.

## Introduction

Tumor cells are not isolated, emerging evidences indicate that carcinogenesis and tumor progression should not be considered as tumor cells autonomous. As a result, the concept of tumor microenvironment (TME) becomes an integrated and essential part for cancers. Besides tumor cells, TME also contains other cell components, which are endothelial cells, fibroblasts, neutrophils, eosinophils, basophils, mast cells, T and B lymphocytes, natural killer cells and antigen presenting cells (for example, macrophages and dendritic cells)[1]. In TME, tumor cells attract or activate many non-tumor cells, which directly (through the release of factors) or indirectly (through the induction of tissue hypoxia or appearance of necrosis) modify the microenvironment. In another hand, TME also promotes tumor progression by stimulating tumor growth, survival, invasion and metastasis.

Macrophages are essential cellular components of the innate immune system, which has been demonstrated recently. These cells play an active role in promoting the carcinogenesis process within TME[2]. Macrophages derive from peripheral blood monocytes, and are recruited into local tumor microenvironment by tumor-derived factors such as cytokines and chemokines. They are “educated” to carry out specific functions to support the growth of tumor cell. Therefore, they are called tumor associated macrophages (TAMs). Some studies found that TAMs participated in tumor proliferation, invasion, metastasis and angiogenesis[3]. Moreover, most clinical studies also revealed that the number of TAMs within TME was correlated with tumor poor prognosis[4].

In most human and mouse cancers, TAMs in TME were M2 polarized population and the phenotype depended on the microenvironment education[5]. Monocytes differentiate into M1 macrophages when they are exposed to granulocyte-macrophage colony stimulating factor (GM-CSF), interferon (IFN)-γ, lipopolysaccharide (LPS) and other microbial agents. In contrast, monocytes differentiate into M2 macrophages when they are exposed to macrophage colony stimulating factor (M-CSF), IL-4, IL-10, IL-13 and some of immuno-suppressive factors. M1 macrophages are also called classically activated macrophages. They have high anti-tumor activity and immuno-stimulatory functions, which produce profuse pro-inflammatory cytokines, such as tumor necrosis factor (TNF)-α and IL-12. M2 macrophages appear as “alternatively” activated macrophages, which secrete high levels of anti-inflammatory cytokines like IL-10. They are characterized by surface expression of macrophage mannose receptor CD206, and are playing the role of tumor progression as indicated previously.

Signal transducers and activators of transcription 3 (STAT3) are among the family of STAT, which mediates several pathways in malignant progression[6,7]. It was found that the activities of STAT3 were elevated in many solid malignancies such as head and neck cancers, breast cancer, prostate cancer, leukemia, lymphomas and multiple myeloma[8–10]. Furthermore, recent studies also showed that the constitutive activation of STAT3 propagated the change from tumor cells to tumor-associated immune cells within TME, such as TAMs, DCs, NK cells, neutrophils and lymphocytes. Crosstalk between these cells could act in concert to facilitate tumor progression. Therefore, in one way, the inhibition of STAT3 could affect tumorigenesis including cell cycle, cell apoptosis, tumor angiogenesis, invasion, metastasis; In another way, blocking STAT3 of adjacent tumor-associated immune cells through a “by-stander effect” could also exert the effect of killing neighboring tumor cell[11]. A growing body of evidences combined with our previous studies demonstrated that “alternatively” activated macrophages (TAMs) had overexpression of pSTAT3. Evidences also supported that STAT3 signaling triggered the crosstalk between tumor cells, TAMs were crucial for the regulation of malignant progression. In this regard, our hypotheses are 1) STAT3 overexpression in TAMs could be considered as a crucial switch that trigger deregulation between tumor cells and TAMs; 2) STAT3 inhibition could re-educate TAMs to transfer from M2 to M1 in order to control the growth, invasion, angiogenesis, and metastasis of tumor cells.

Hydrazinocurcumin (HC), a synthetic analogue of curcumin (the active constituent of turmeric, a polyphenolic compound), improves its water solubility, stability, cell permeability and bioavailability with superior pharmacological activity compared to curcumin[12]. In our previous study, we demonstrated that HC was an effective inhibitor of STAT3 phosphorylation. It could down-regulate an array of STAT3 downstream targets that contributed to the suppression of cell proliferation, loss of colony formation, depression of cell migration and invasion as well as induction cell apoptosis in vitro[13]. Therefore, in this study, HC was employed to suppress STAT3 activity, and re-educate TAMs to change from M2 to M1. Moreover, we followed the Legumain-targeting strategy used in our previous studies [14] and used RR-11a-coupled liposomal nanoparticles (NPs) encapsulating HC to reduce nonspecific accumulation in the reticuloendothelial system and to enhance targeting capability in solid tumors[14,15].

## Materials and Methods

### Ethics Statement

The care of laboratory animal and the animal experimental operation were carried out in strict accordance with Chongqing Management Approach of Laboratory Animal (Chongqing government order NO.195). The protocol was approved by the Ethics Committee of Chongqing Medical University (Reference Number: CQMU 2010-26). All surgery was performed under sodium pentobarbital anesthesia, and all efforts were made to minimize suffering.

### Cells and animals

4T1 murine breast tumor cell line was provided kindly by Dr. Rong Xiang, School of Medicine, Nankai University (Tianjin, China), and RAW264.7 mouse monocyte/macrophage cell line was a gift from Dr. Yangan Wen. These cell lines were sourced from American Type Culture Collection (ATCC) (Rockville, MD, USA). Cells were kept in Dulbecco’s Modified Eagle Medium containing 10% fetal bovine serum, penicillin (100U/ml) and streptomycin (100µg/ml). Co-culture condition with transwell inserts (porous polycarbonate membrane filters with 0.4µm pore) and 6-well plastic culture plates were used for 4T1-RAW264.7 co-culture. Female BALB/c mice, 6-8 weeks old, were purchased from Center of Laboratory Animals, Chongqing Medical University (Chongqing, China).

### HC-NPs and Legumain-targeted HC-NPs preparation

DOPE, DOPC, Cholesterol, DOPE-PEG and HC were mixed together in molar ratios of 1:1:1:0.16:0.9 with stirring and then rotavaporate at 40^o^C. The dried lipid film plus PBS were vortexed for 2~3 minutes and agitated for a minimum of 6 hours. Liposomes were sonicated for 2-3minutes to in a bath sonicator at room temperature to produce multilamellar vesicles (MLV). MLVs were sonicated again in the probe sonicator for 1 min (25W output power) to produce small unilamellar vesicles (SUVs). The solution was pressure filtered in sequence through 220nm and 100 nm nucelopore polycarbonate membranes to obtain liposome nanoparticles of 100nm. The preparation of control nanoparticles was as same as HC-NPs without adding HC.

Legumain-targeted HC-NPs were prepared as previously reported [16]: 1ml Carproylamine PE (25mg/ml), 16.3mg Legumain inhibitor, 3µl Triethylamine (TEA) were mixed together by stirring at room temperature for 24 hours, the exact mass of the peptide lipid conjugates were verified as 30mM by mass spectroscopy. DOPE, DOPC, Cholesterol, DOPE-PEG, HC and peptide-lipid were mixed together in molar ratios of 1:1:1:0.16:0.9:0.17 by stirring and then rotavaporate at 40^o^C. The rest of the procedures were in accordance with HC-NPs.

### Flow cytometry

M1, M2 macrophage markers were determined by staining RAW264.7 cells treated with HC-NPs, before or after co-cultured with FITC-labeled anti-CD86, anti-CD206 in combination with PE-labeled anti-IL-10, anti-IL-12, anti-TGF-β, followed by FACS analyses. All antibodies were purchased from Biolegend (San Diego, CA). IL-10, IL-12 or TGF-β release at the intracellular level was determined with PE-labeled anti-IL-10, anti-IL-12, anti-TGF-β. Cells were fixed with 4% paraformaldehyde, permeabilized with permeabilization wash buffer (BD Biosciences), and subsequently stained with PE-labeled antibodies to detect intracellular expression of IL-10, IL-12, TGF-β.

### Western blot analysis

For Western blot, protein from cancer cells or macrophages lysates was subjected to SDS-PAGE and transferred to PVDF membrane. Blots were probed with pSTAT3 (Tyr 705) antibody (Cell Signaling Technologies, USA), with STAT3 antibody (B.D, USA), with MMP-9, MMP-2 antibodies (Bioword, USA), with VEGF, Bcl-2, β-actin antibodies (Santa Cruz Biotechnology, USA), with PARP, Caspase 3 antibodies (Beyotime, China). Membranes were analyzed using Enhanced Chemiluminescence (ECL) detection system (VIAGENE, USA).

### MTT cell proliferation inhibition assay

E-RAW264.7 cells were seeded in 96-well plates at a density of 3000 cells per well. Different concentrations of NPs or HC-NPs (9-36µM) were added in triplicate to the plates in the presence of 10% FBS. The cells were incubated at 37^°^C for different period (6 h, 12 h, 24h), and then 25µL MTT (Sigma, USA) was added to each sample; after 4 hours, 100µL DMSO (Sigma, USA) was added to each well. The absorbance was detected at 490nm, and the viability of the untreated cells was arbitrarily set at 100% compared with the viability of NPs or HC-NPs -treated cells.

### Cell cycle and cell apoptosis analysis

Cell cycle phase was determined by fluorescence-activated cell sorting analysis. 4T1 cells were seeded in six-well plates at a concentration of 5×10^5^ per well, then co-cultured with RAW264.7 cells (1 × 10^5^ per well into upper chamber, pre-treated with NPs or HC-NPs for 12 h) for 48 h, cells were collected, and sorted using a Flow cytometric (Bekman coulter, USA). Cells of apoptosis analysis were treated as described previously. After collection, cells were washed by PBS 3 times, and then resuspended in 0.5 ml PBS. Cells were stained with Annexin V and propidium iodide (PI) in the presence of 100 mg/ml RNAse and 0.1% Triton X-100 for 30 min at 37^°^C. Flow cytometric analysis was performed using a fluorescence-activated cell sorter.

### Migration and invasion assays

Tumor cell migration and invasion assays were performed using Transwell system (Millipore, USA) with 8 µm-pore polycarbonate filter membrane. For migration assay, the upper chamber was seeded with 1 × 10^4^ 4T1 cells and inserted into the lower chamber which filled with DMEM contained 15% FBS. After incubation for 8 h, the cells on the interior of upper chamber were removed, and the polycarbonate membranes were stained with 0.1% crystal violet for 10 min. The number of migrating cells was counted in five randomly selected fields under microscope. The procedure of invasion assay was similar to the migration assay except the addition of Matrigel (Sigma, USA) into upper chamber and incubation for 20 h.

### Tumor cell challenge and therapeutic treatment

Mammary fat pads of female BALB/c mice were co-injected with 1 × 10^6^ 4T1 cells and 1 × 10^6^ E-RAW264.7 cells. 10 days later, mice received 5 IV injections of PBS, NPs, legumain-targeting HC-NPs (1 mM), free HC (100 µM) at 3-day intervals. The experiment was terminated at day 25, some mice were sacrificed at day 30, and tumor weights were determined and tissues subjected to histological analysis. Other mice were kept to observe mouse survival rates until 60 days. Ki-67, TUNEL, CD31 immunohistochemical staining of tumor tissue sections was performed according to the manufacturer’s protocol. H＆E staining of lung tissue sections demonstrated pulmonary metastasis in models.

### Statistical analysis

Statistical significance was determined using the Student’s t test and Graph Pad Prism software. Data were expressed as the mean ± SD. p value < 0. 05 was considered significant.

## Results

### Polarization of macrophages from M1 to M2 after co-cultured with 4T1 breast cancer cells

**Figure 1:**
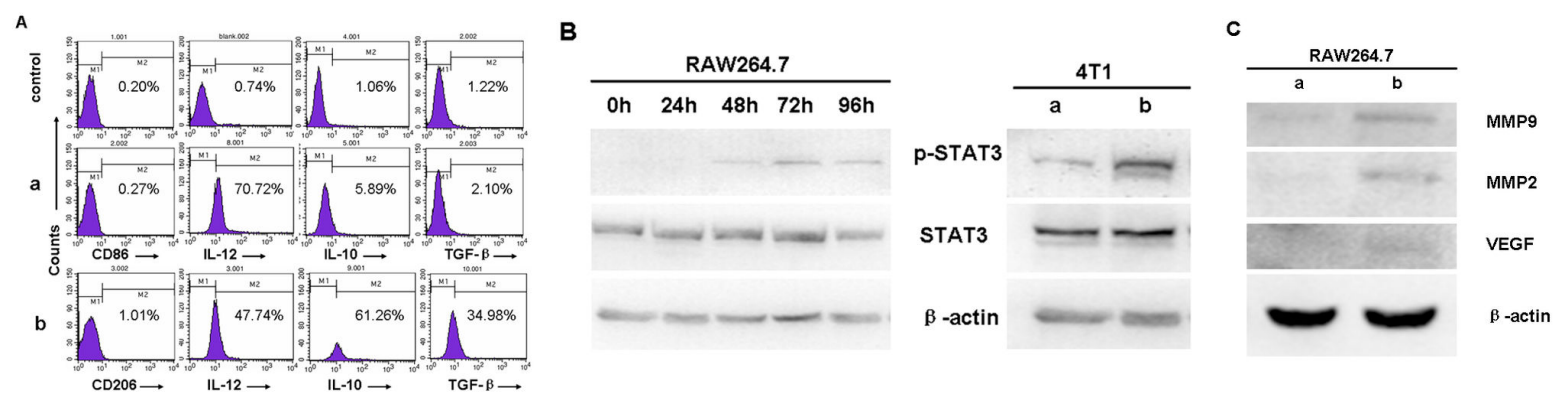
Polarization of macrophages from M1 to M2 after co-cultured with 4T1 breast cancer cells. Flow cytometry demonstrated upregulation of M2 macrophages intracellular markers on RAW264.7 after co-cultured with 4T1 cells. a. before co-culture; b. after co-culture. CD86, CD206, IL-10, IL-12, TGF-β expression on macrophages and their isotype control was evaluated separately by FACS. Data are representative of 3 separate experiments. (B) Increased p-STAT3 expression in macrophages after co-cultured with 4T1 cells (1×10^6^). a. before co-culture; b. after co-culture. (C) Increased expression of MMP2, MMP9 and VEGF of RAW264.7 cells after co-culture; a. before co-culture; b. after co-culture.

To test whether tumor cells could educate macrophages phenotype transformation from M1 to M2, RAW264.7 mouse monocyte/macrophages were co-cultured with 4T1 mouse breast cancer cells *in vitro*. Intra-cellular cytokines of the educated RAW264.7 cells (E-RAW264.7) co-cultured with 4T1 cells for 48-72 hours were detected with flow cytometry. As anticipated, E-RAW264.7 cells showed a M2-like phenotype, IL-10^high^, IL-12^low^, TGF-β^high^; however, the cells did not demonstrate obvious rise of CD206 which was one of the surface antigen makers of M2 macrophages (Figure 1A). Simultaneously, up-regulation of tyrosine phosphorylation of STAT3 (p-STAT3, Tyr705) was investigated in both macrophages and tumor cells in a manner of time-dependence (Figure 1B). MMP9, MMP2 and VEGF, three downstream targets of STAT3, also increased in E-RAW264.7 cells after co-cultured with 4T1 cells (Figure 1C).

### HC-NPs “re-educate” TAMs phenotype from M2 to M1 following STAT3 suppression

**Figure 2:**
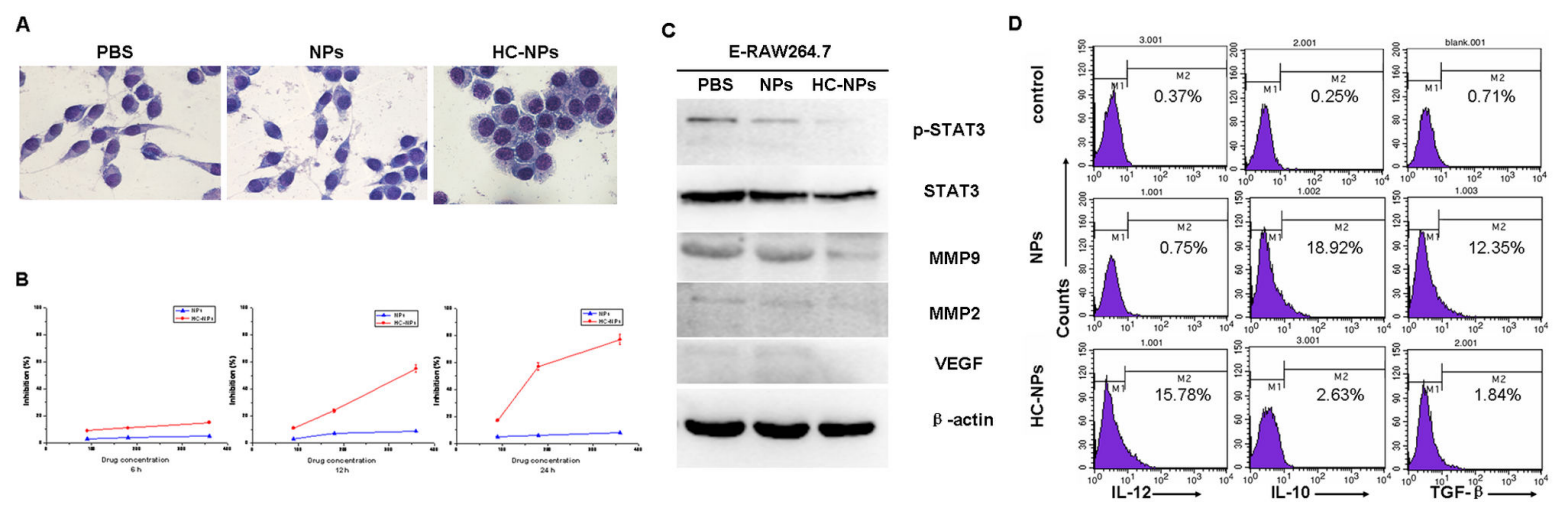
HC-NPs “re-educate” TAMs phenotype from M2 to M1 following STAT3 suppression. (A) HC-NPs affected morphological changes of E-RAW264.7 cells; PBS, NPs, HC-NPs represented cells were treated with PBS, NPs, 180µM HC-NPs for 12 hours respectively (Wrights staining, Magnification×100.0). (B) MTT demonstrated HC-NPs inhibited growth of E-RAW264.7 cells in a manner of time-concentration-dependence, after treated 6h, 12h, 24 h. (C) Decreased p-STAT3, MMP-2, MMP-9 and VEGF protein expression of E-RAW264.7 cells after treated with HC-NPs. PBS, NPs, HC-NPs represented cells were treated with PBS, NPs, 180µM HC-NPs for 12 h respectively. (D) FACS demonstrated downregulation of M2macrophages intracellular markers in RAW264.7 after treated with HC-NPs, NPs.

HC was taken as an effective STAT3 activation inhibitor, which was enveloped with liposomal NPs. 18µM of HC-NPs was used to treat E-RAW264.7 cells for 12 hours on basis of MTT analysis (Figure 2B). HC-NPs treated E-RAW264.7 cells had great morphological changes, and the cells became round and there were a few vacuole in the cytoplasm compared with control groups (Figure 2A). Importantly, the results showed that HC-NPs could revert E-RAW264.7 phenotype from M2 to M1, and the cells were named as re-educated RAW 264.7 (RE-E-RAW264.7) which exhibited M1 phenotype, IL-10^low^, IL-12^high^, TGF-β^low^ (Figure 2D), along with down-regulation of p-STAT3, MMP9, MMP2, VEGF (Figure 2C).

### “Re-educated” macrophages affected 4T1 cell proliferation, cell cycle, cell apoptosis, migration and invasion

**Figure 3:**
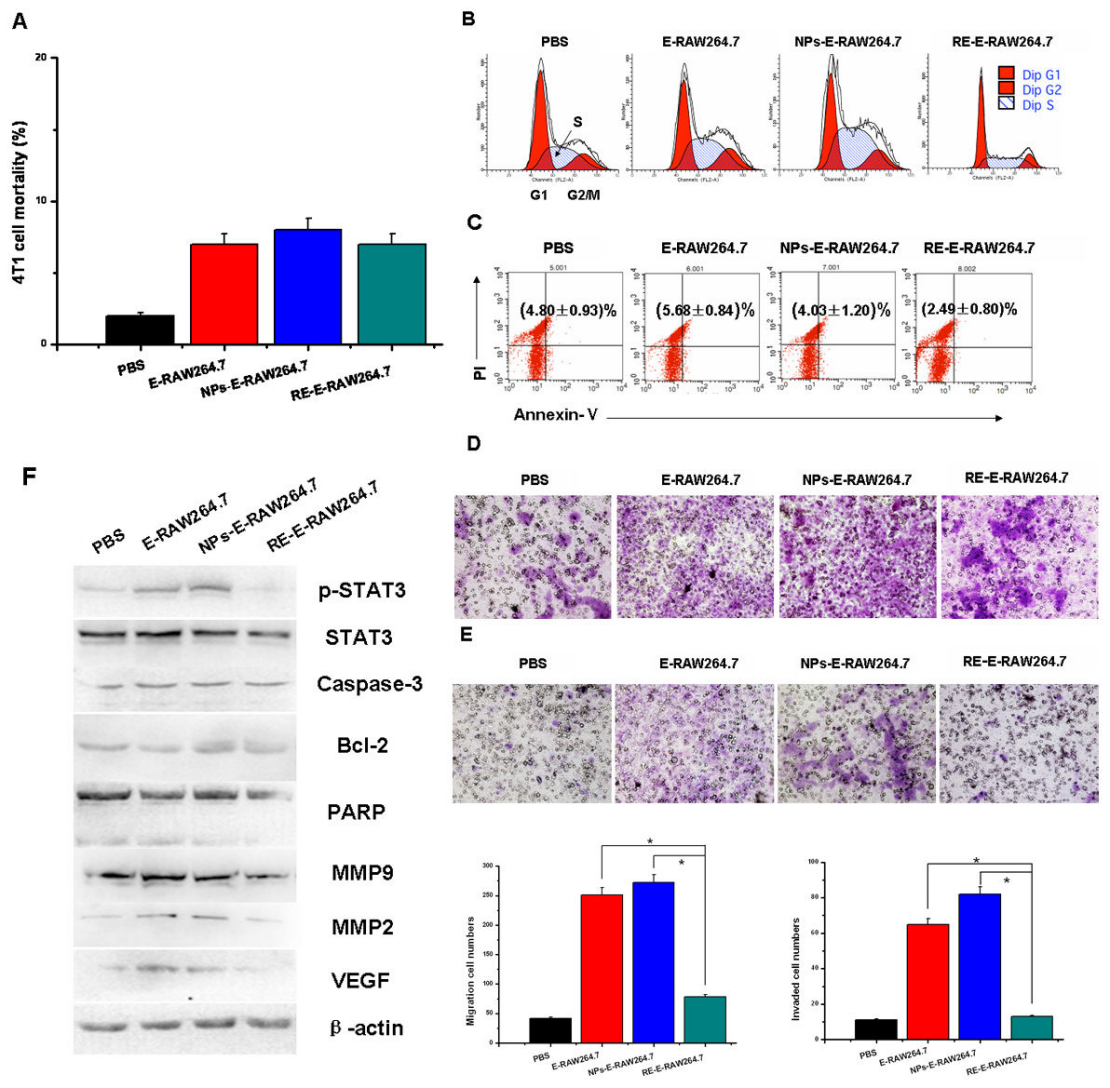
“Re-educated” macrophages affected 4T1 cell proliferation, cell cycle, cell apoptosis, migration and invasion. (A) Trypan blue staining demonstrated mortality of 4T1 cells incubated with PBS, co-cultured with E-RAW264.7, E-RAW264.7 treated with plain NPs (NPs-E-RAW264.7) or E-RAW264.7 treated with 18 µM HC-NPs (RE-E-RAW264.7) for 12 hours respectively. (B) (C) Flow cytometry detected cell cycle and cell apoptosis of 4T1 cells incubated with PBS, co-cultured with E-RAW264.7, NPs-E-RAW264.7, RE-E-RAW264.7 for 12 hours respectively. X Parameter, Annexin-v (Log); Y Parameter, PI (Log). Data are representative of 3 separate experiments. (D) (E) Migration and invasion of 4T1 cells significantly decreased after co-cultured with RE-E-RAW264.7. Transwell migration and invasion assays, cells were stained with 0.1% crystal violet solution and analyzed with ImageJ. Original magnification: × 200. (F) The “re-educated” macrophages affected p-STAT3, STAT3, Caspase-3, Bcl-2, PARP, MMP-9, MMP-2 and VEGF protein expression of 4T1 cells after RE-E-RAW264.7/4T1 co-culture.

To further investigate whether RE-E-RAW264.7 cells would affect tumor cells progression, we co-cultured 4T1 cells with E-RAW264.7 (M2-like phenotype) or RE-E-RAW264.7 cells (M1-like phenotype), and PBS group, empty NPs group were used as control. 4T1 cells exhibited different tumorigenicity: E-RAW264.7 increased cell number in the S phase, promoted migration and invasion capabilities of 4T1 cells, and increased MMP-9, MMP-2, VEGF expression. In the contrast, the effect of RE-E-RAW264.7 was considerably opposite. Trypan blue exclusion assay and cell apoptosis assay showed that RE-E-RAW264.7 cells had no effect on 4T1 cells mortality and apoptosis (P>0.05) (Figure 3A, 3C). But it was notably found that RE-E-RAW264.7 cells significantly decreased 4T1 cell number in the S phase (38.24±2.21) % compared to the PBS group (45.21±3.72) % and NPs group (53.79±3.55) % (P=0.0004 and P<0.001) (Figure 3B). Moreover, migration and invasion of tumor cells were detected by transwell co-culture system, and the data indicated that RE-E-RAW264.7 cells was less effective in the induction of 4T1 cells migration and invasion (Figure 3D, 3E). Since the Expression of p-STAT3 protein as well as its downstream targets might account for 4T1 cells reduction of tumorigenicity, we discovered that the expression of p-STAT3, MMP9, MMP2 and VEGF were related to the migration and invasion of tumor cells; while the expression of caspase3, Bcl-2, PARP were related to tumor cells apoptosis. It was found that p-STAT3, MMP9, MMP2, VEGF decreased, while caspase3, Bcl-2, PARP had no significant change (Figure 3F).

### Legumain-targeting HC-NPs inhibited breast tumor growth and prolonged tumor-bearing mice survival when 4T1 co-injected with RAW264.7 into mice fat pad

**Figure 4:**
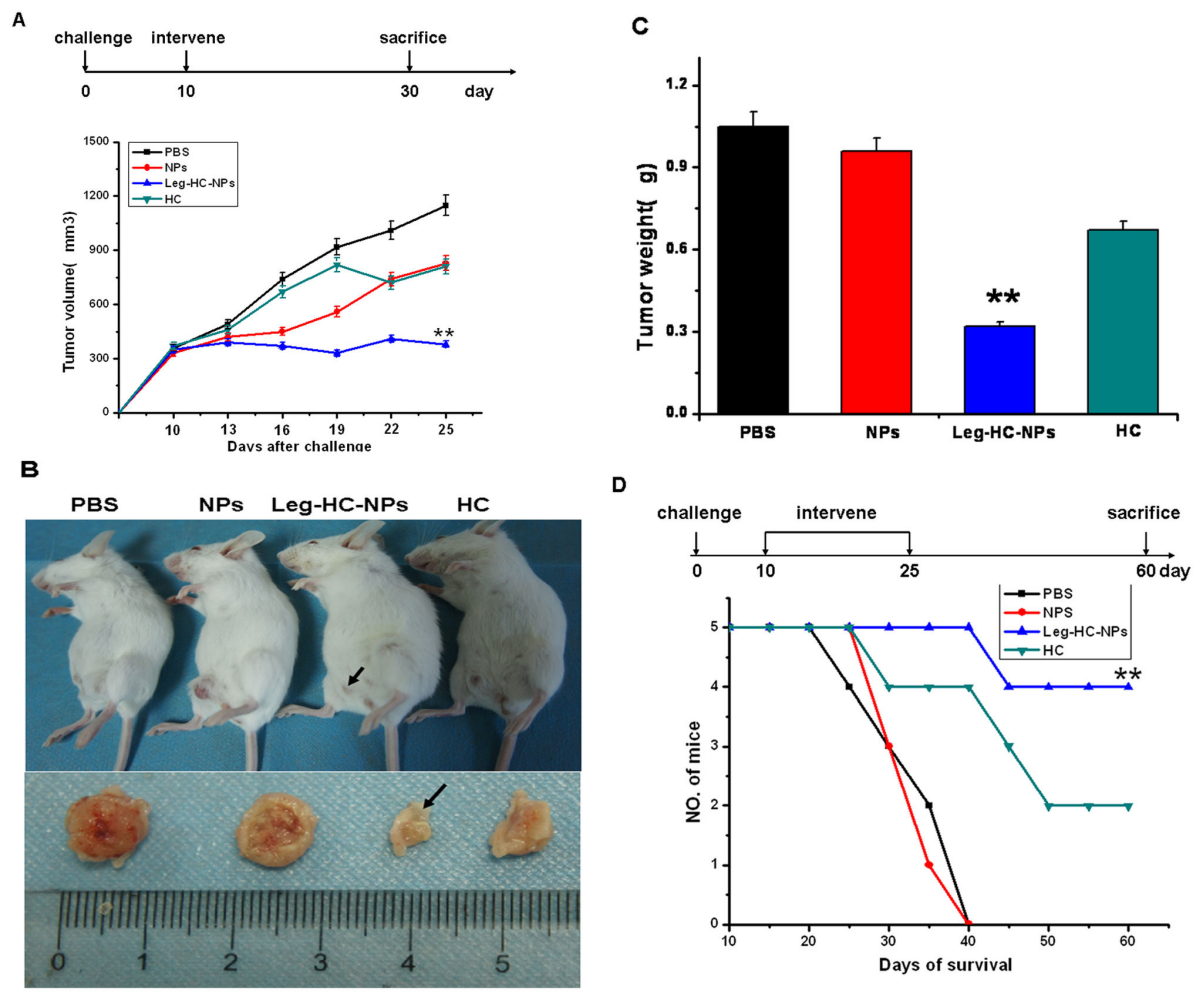
Legumain-targeting HC-NPs inhibited breast tumor growth and prolonged tumor-bearing mice survival time when 4T1 co-injected with RAW264.7 into mice fat pad. Groups of female BABLB/C mice (n = 5) were co-injected with 1×10^6^ 4T1 tumor cells and 1×10^6^ E-RAW264.7 cells in the mammary fat pad and 10 days later, give 5 IV injections on days 10, 13, 16, 19, 22, 25 with PBS, NPs, Legumain-targeting HC-NPs (Leg-HC-NPs) and free HC respectively. (A) Tumor volume after treatment with Leg-HC-NPs, HC or controls (PBS or NPs). (B) The difference in tumor size at day 30 after 5 doses of treatment when mice were sacrificed. (C) The measurement of primary tumor wet weight. (D) The difference of survival time between Leg-HC-NPs treatment and control groups, survival plots stood for 5 mice in each group.

To determine the effect of HC-NPs on the tumor growth *in vivo*, 4T1 cells were co-injected with RAW 264.7 cells into BALB/c female mice fat pad to constitute mice breast cancer transplanted tumor models. When tumor volume reached approximately 350 mm^3^ on day 10, mice were injected with PBS, NPs, Legumain-targeting HC-NPs (Leg-HC-NPs) (1 mM) and HC (100 µM) respectively at 3 days intervals within 15 days. As shown in Figure 4A, within 25 days, the size of subcutaneous tumor in Leg-HC-NPs treated group was significantly smaller than that of controls (P < 0.05). On day 30, some of the mice were sacrificed, primary tumor was surgically excised, and it was found that treatment with Leg-HC-NPs resulted in a distinct decrease in tumor weight when compared with the controls (Figure 4B, 4C). Life-span curve also indicated that 80% (4/5) of the mice in Leg-HC-NPs group survived for more than 2 months. In contrast, mice in the control groups all died within 40 days. In addition, low survival rate (40%, 2/5) in HC group might result from the low bioactivity of HC compared with Leg-HC-NPs (Figure 4D).

### Legumain-targeting HC-NPs promoted tumor cells apoptosis and inhibits tumor cells proliferation, angiogenesis and pulmonary metastasis in vivo

**Figure 5:**
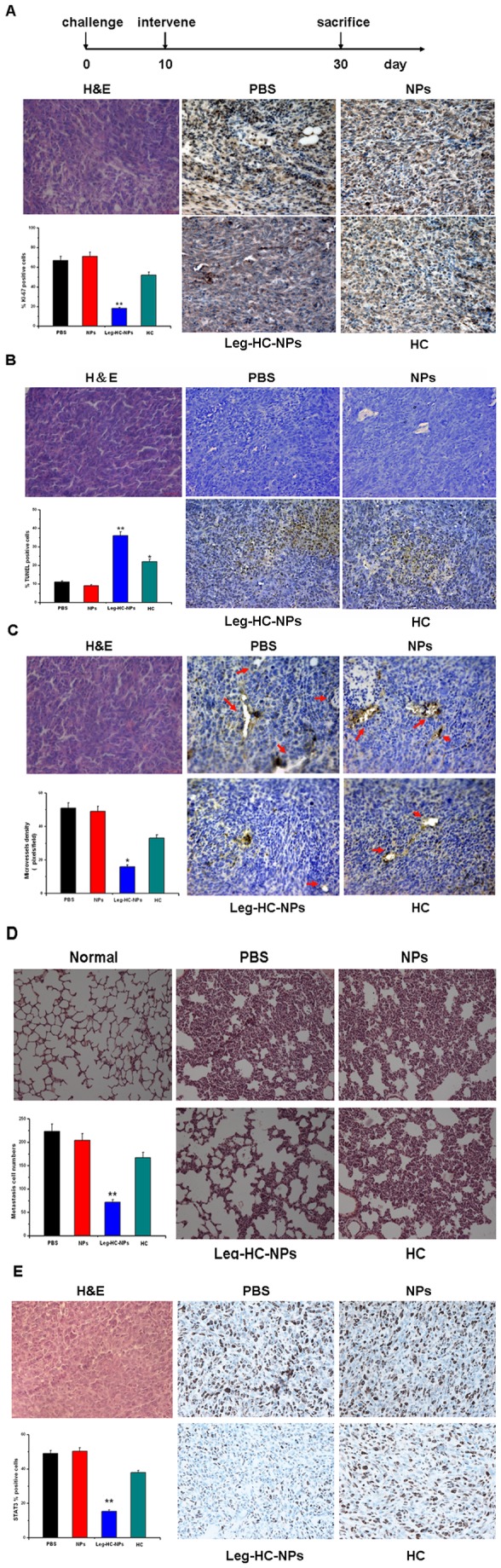
Legumain-targeting HC-NPs promoted tumor cells apoptosis and inhibits tumor cells proliferation, angiogenesis and pulmonary metastasis in vivo. Tumor bearing mice were sacrificed at day 30 after 5 IV injections. (A) Expression of ki-67 in tumor sections was evaluated by immunohistochemistry (IHC). Original magnification: × 400. (B) TUNEL staining visualized and quantified the percentage of apoptotic tumor cells in tumor sections. Original magnification: × 400. (C) CD31-positive microvessel density was evaluated by counting at × 400 magnification. Arrows show positively-stained microvessels. (D) H&E staining demonstrates pulmonary metastasis in vivo. Original magnification: × 200. (E) Expression of STAT3 in tumor sections was evaluated by IHC. Original magnification: × 400.

To further examine the role of Leg-HC-NPs in inhibition of tumor progression in murine models, STAT3 and cells proliferation, apoptosis and angiogenesis associated factors were analyzed in situ immunohistocheminstry. It was revealed in Figure 5A, 5E that the expression of Ki-67 which was investigated positive in proliferative tumor cells was strikingly reduced in Leg-HC-NPs treated group, and STAT3 was also reduced in this group. And the result from TUNEL immunohistochemical analysis showed that the Leg-HC-NPs treated mice presented an increase in percentage of apoptotic cells when compared with that of the controls (Figure 5B). CD31-positive microvessel density were examined in different groups (Figure 5C), and it was verified that Legumain-targeted HC-NPs could reduce angiogenesis in tumor progression. Furthermore, pulmonary metastasis was investigated by counting tumor cell number in lung section in different group, and metastatic tumor cells in Leg-HC-NPs treated group had a 3-fold reduction when compared with control groups (Figure 5D).

## Discussion

This study employed HC as an inhibitor of STAT3 activation, and demonstrated that HC encapsuled NPs efficiently “re-educated” tumor associated macrophages *in vitro* to transform from M2 to M1. These transformations regulated the crosstalk between tumor cells and TAMs, subsequently controlled the proliferation, migration and invasion of tumor cells when re-educated macrophages co-cultured with 4T1 cells *in vitro*, and thereby suppressed the tumor growth, angiogenesis and metastasis *in vivo*.

In our study, firstly, RAW264.7 macrophages were co-cultured with 4T1 breast cancer cells in vitro to investigate whether tumor cells could educate macrophages phenotype transformation from M1 to M2. It was verified that 4T1 could successfully “educated” macrophages to perform M2 phenotype (IL-10^high^, IL-12^low^, TGF-β^high^) [17–19], and p-STAT3, MMP2, MMP9, VEGF were also increased. Opposing to what we expected, E-RAW264.7 cells did not show significant increased CD206 level which was one of surface antigen makers of M2 macrophages under our experimental conditions. It might be due to the differences between real tumor microenvironment *in vivo* and the co-culture system model used in this study. The former microenvironment might contain more multiple elements within TME, such as fibroblasts, vascular endothelium cells, Treg cells and immature DC cells as well as crosstalks, contacts between these cells, which could more effectively educate macrophages; while RAW264.7/4T1 co-culture system in vitro did no completely mimic the TME *in vivo*, this might interpret why the CD206 surface antigen marker didn’t change.

Subsequently, HC-NPs was used to re-educate E-RAW264.7 cells (M2-like phenotype) for 12 hours; Re-educated RAW264.7 cells (RE-E-RAW264.7) exhibited M1-like phenotype (IL-10^low^, IL-12^high^, TGF-β^low^) (Figure 2D), along with down-regulation of p-STAT3, MMP9, MMP2, VEGF (Figure 2C). The results indicated that HC-NPs could re-educate E-RAW264.7 phenotype to transform from M2 to M1, and our results implied that STAT3 might be a key contributor for macrophages polarization.

The crosstalk between TAMs and tumor cells were considered to play a crucial role in the tumor progression. Ablation of macrophage function or their infiltration into experimental tumors inhibits cancer growth and metastasis[20–23]. For example, the antineoplastic drug Yondelis has a selective cytotoxicity on TAMs, thereby significantly reduces their production of IL-6 and CCL2, which conversely contribute to growth suppression of inflammation-associated human tumors[24]. Another similar example was provided by a biphosphonate compound, zoledronic acid, which could inhibit tumor metalloproteinase activity and diminish the association of VEGF with its tyrosine kinase receptors on proliferating endothelial cells by suppressing MMP-9 secretion of TAMs[16]. Researchers also found that interrupting STAT3 signaling in TME induced tumor cells to produce soluble factors capable of mediating bystander tumor cell killing. Based on these reports, we discovered how RE-E-RAW264.7 controlled the proliferation, apoptosis, migration, invasion and other malignant biological functions of breast cancer cells. In our study, co-culture with “educated” or “re-educated” macrophages resulted in different tumorigenicity of 4T1 breast tumor cells: “M2-like” macrophages (E-RAW264.7) increased tumor cell number in the S phase, promoted migration and invasion capabilities of 4T1 cells, and increased MMP-9, MMP-2, VEGF expression. In the contrast, HC-NPs treated macrophages (RE-E-RAW264.7) demonstrated opposite effect of “M2-like” macrophages, especially the induction of 4T1 cells migration and invasion. Nevertheless, neither “educated” nor “re-educated” macrophages-treated group demonstrated effect on 4T1 cells apoptosis, and this indicated RE-E-macrophages might not affect apoptosis of tumor cells *in vitro*.

As reported in our previous study, Legumain-targeting NPs enhanced solid-tumor penetration compared with non-targeting NPs; treatment of tumor-bearing mice with RR-11a-coupled NPs encapsulating doxorubicin resulted in improved tumor selectivity and drug sensitivity, leading to completely inhibition of tumor growth. Based on the results, we took following Legumain-targeting strategy [16], RR-11a-coupled liposomal nanoparticles (NPs) encapsulating HC were used to reduce nonspecific accumulation in the reticuloendothelial system, and to enhance targeting ability in solid tumors. It was shown that in our study, when compared with free HC, Leg-HC-NPs increased tumor sensitivity to drug doses, and more effectively inhibited breast tumor growth, angiogenesis and pulmonary metastasis, and in turn prolonged mice life span. Furthermore, the remarkable findings showed that Leg-HC-NPs could induce apoptosis of tumor cell *in vivo* by TUNEL assay, and there was difference in inhibition of HC-NPs *in vitro* or *in vivo*. Moreover, the result demonstrated that, to some extent, Leg-HC-NPs eliminated systemic toxicities induced by high doses of HC in mice, which led to fester of caudal vein and high mortality after 5 IV injections within 15 days. The findings supported that Leg-HC-NPs were more capable for clinical application compared with free HC.

In sum, Legumain-targeting liposomal nanoparticles (NPs) encapsulating HC were employed to suppress STAT3 activity and “re-educate” TAMs, and the results indicated that through inhibition of STAT3, HC-NPs was able to reverse TAMs phenotype and could regulate their crosstalk between tumor cells and TAMs. However, because HC had effects on modulation the activation of various transcription factors and multiple signaling pathways [25,26], STAT3 specific inhibitor [27,28] or siRNA against STAT3 should be included in future study to elucidate that STAT3 might be a key contributor for macrophages polarization.
