# Expression of Concern: Hydrazinocurcumin Encapsuled Nanoparticles "Re-Educate" Tumor-Associated Macrophages and Exhibit Anti-Tumor Effects on Breast Cancer Following STAT3 Suppression

**DOI:** 10.1371/journal.pone.0220672

**Published:** 2019-07-29

**Authors:** The PLOS ONE Editors

After publication of this article [1], concerns were raised about Figs 1 and 3:

- In Fig 1B, lanes 1–2 of the RAW264.7 β-actin panel appear similar to lanes 1–2 of the 4T1 β-actin panel.
- Lanes 1, 2 of the 4T1 STAT3 panel in Fig 1B appear similar to lanes 1, 2 of the STAT3 panel in Fig 3F, although the lanes in the two figures represent different experimental conditions.

The authors confirmed that the same β-actin data were knowingly reported in the two panels of Fig 1B and were not considered essential in supporting the experiment’s results. The authors also claimed that the STAT3 duplication resulted from a sample labeling error.

The authors provided replication data for the β-actin and STAT3 panels of Figs 1B and 3F, but did not provide p-STAT3 data from the same replication experiments. The journal received no further clarification around the replication experiments or the underlying data for other results in this article. As such, the data provided do not fully clarify the issues pertaining to the overall results.

In light of the unresolved questions around the control data for experiments reported in Figs 1B and 3F and the availability of the data for the results reported in this article, the *PLOS ONE* Editors issue this Expression of Concern.
